# Comparison of different gene addition strategies to modify placental derived-mesenchymal stromal cells to produce FVIII

**DOI:** 10.3389/fimmu.2022.954984

**Published:** 2022-12-15

**Authors:** Ritu M. Ramamurthy, Martin Rodriguez, Hannah C. Ainsworth, Jordan Shields, Diane Meares, Colin Bishop, Andrew Farland, Carl D. Langefeld, Anthony Atala, Christopher B. Doering, H. Trent Spencer, Christopher D. Porada, Graça Almeida-Porada

**Affiliations:** ^1^ Fetal Research and Therapy Program, Wake Forest Institute for Regenerative Medicine, Winston Salem, NC, United States; ^2^ Department of Biostatistics and Data Sciences Wake Forest School of Medicine, Winston Salem, NC, United States; ^3^ Department of Pediatrics, Aflac Cancer and Blood Disorders Center, Children’s Healthcare of Atlanta, Emory University, Atlanta, GA, United States; ^4^ Department of Medicine, Hematology and Oncology, Wake Forest School of Medicine, Winston Salem, NC, United States

**Keywords:** CRISPR/Cas, lentiviral (LV) vector, cell therapy, gene therapy, FVIII, hemophilia A, placental-derived mesenchymal stromal cells

## Abstract

**Introduction:**

Placenta-derived mesenchymal cells (PLCs) endogenously produce FVIII, which makes them ideally suited for cell-based fVIII gene delivery. We have previously reported that human PLCs can be efficiently modified with a lentiviral vector encoding a bioengineered, expression/secretion-optimized fVIII transgene (ET3) and durably produce clinically relevant levels of functionally active FVIII. The objective of the present study was to investigate whether CRISPR/Cas9 can be used to achieve location-specific insertion of a fVIII transgene into a genomic safe harbor, thereby eliminating the potential risks arising from the semi-random genomic integration inherent to lentiviral vectors. We hypothesized this approach would improve the safety of the PLC-based gene delivery platform and might also enhance the therapeutic effect by eliminating chromatin-related transgene silencing.

**Methods:**

We used CRISPR/Cas9 to attempt to insert the bioengineered fVIII transgene “lcoET3” into the AAVS1 site of PLCs (CRISPR-lcoET3) and determined their subsequent levels of FVIII production, comparing results with this approach to those achieved using lentivector transduction (LV-lcoET3) and plasmid transfection (Plasmid-lcoET3). In addition, since liver-derived sinusoidal endothelial cells (LSECs) are the native site of FVIII production in the body, we also performed parallel studies in human (h)LSECs).

**Results:**

PLCs and hLSECs can both be transduced (LV-lcoET3) with very high efficiency and produce high levels of biologically active FVIII. Surprisingly, both cell types were largely refractory to CRISPR/Cas9-mediated knockin of the lcoET3 fVIII transgene in the AAVS1 genome locus. However, successful insertion of an RFP reporter into this locus using an identical procedure suggests the failure to achieve knockin of the lcoET3 expression cassette at this site is likely a function of its large size. Importantly, using plasmids, alone or to introduce the CRISPR/Cas9 “machinery”, resulted in dramatic upregulation of TLR 3, TLR 7, and BiP in PLCs, compromising their unique immune-inertness.

**Discussion:**

Although we did not achieve our primary objective, our results validate the utility of both PLCs and hLSECs as cell-based delivery vehicles for a fVIII transgene, and they highlight the hurdles that remain to be overcome before primary human cells can be gene-edited with sufficient efficiency for use in cell-based gene therapy to treat HA.

## Introduction

Hemophilia A (HA) is an X-linked inherited disorder caused by mutations in the gene encoding clotting factor 8 (FVIII), which results in defective/absent coagulation activity. The estimated frequency is 1 in 5000 live male births, and approximately 400,000 people live with HA worldwide (1). HA can be categorized into three degrees of severity depending on the levels of FVIII: severe (<1%), moderate (1-5%), and mild (6-30%). More than half (60%) the people diagnosed with HA are of the severe type (2). Severe HA is characterized by spontaneous bleeds, debilitating hemarthrosis, and life-threatening intracranial hemorrhages. Current treatments for HA primarily include regular infusions of FVIII protein twice a week for the duration of life. Not only is the treatment lifelong, but also expensive and not accessible to ~75% of HA patients (3). Currently, the lifetime cost estimate to treat a patient with HA is ~$20 million (4). Moreover, 30% of the patients who receive the treatment develop inhibitors against the infused protein, rendering the treatment ineffective (5).

Since HA is a monogenic disease, gene therapy represents a promising therapeutic approach for achieving lifelong phenotypic/clinical correction. To-date, over 3700 distinct mutations within the F8 gene have been identified in HA patients (6). Since the ideal therapy would be “universal” and benefit all HA patients, a “gene addition” approach to knock-in a functional fVIII transgene would be preferable to attempting to correct the mutation *via* gene-editing, which would require gRNA and homology arms specific to each patient’s unique mutation. Currently, there are at least 10 ongoing “gene addition” clinical gene therapy trials for HA, with the majority using Adeno-associated virus (AAV) (7–9). While results to-date have been promising, major challenges such as immunologic barriers and the need for very high vector dose to achieve a clinically meaningful rate of transduction remain (7–13). In addition, the vast majority of AAV vector genomes remain episomal following transduction (14, 15), raising concerns over the duration of phenotypic correction that can be achieved with this approach. Recent data emerging from ongoing clinical trials showing that plasma FVIII levels are decreasing with time post-treatment validate this concern (10–13).

An attractive alternative to directly injecting viral vectors to achieve “gene addition” that is gaining momentum to achieve phenotypic correction of HA (16) is to genetically modify appropriate cells and to then use these engineered cells as vehicles to carry the therapeutic transgene. Such an approach allows the inclusion multiple safeguards during manufacturing that are not possible with direct vector injection, such as determination of vector copy number, quantitation of transgene expression, identification of genomic integration sites, and thorough tumorigenicity/toxicology testing. For such an approach to work for HA, the cellular vehicle must be carefully selected to ensure that the large and complex FVIII protein can be efficiently synthesized, post-translationally processed, and secreted without inducing cell stress response, while maintaining its procoagulant activity (17, 18).

Among the myriad putative cellular vehicles, one could consider, human placenta-derived mesenchymal stromal cells (PLCs) possess a host of unique biological and immunological properties that make them ideally-suited as an off-the-shelf product for cell-based therapies and for gene/drug delivery (19–21). One feature of particular relevance as a cellular platform for treating HA is that PLCs constitutively produce FVIII mRNA, protein, and procoagulant activity (22), establishing that they possess all the requisite machinery to produce, process, and secrete this complicated protein and preserve its functionality. Moreover, PLCs also endogenously produce von Willebrand factor (vWF) (22), which serves as FVIII’s carrier protein *in vivo*, dramatically prolonging its biological half-life (23–25), and preventing its uptake and presentation by antigen-presenting cells, thereby reducing its potential immunogenicity (26, 27). Based on these promising traits, we recently investigated the ability of human PLCs to serve as cellular vehicles for delivering a fVIII transgene. The complexity of the FVIII protein has always been a hurdle to developing gene therapy for HA (8, 28–30). However, the past decades have seen remarkable progress in bioengineering techniques, like codon optimization, that have been shown to enhance FVIII protein translation *in vitro* and in clinical trials (31, 32). We established that transducing PLCs with a lentiviral vector encoding a fVIII transgene that was >92% human, but which was codon optimized and bioengineered to include porcine sequence elements that have been shown to enhance post-translational processing and secretion of FVIII (22, 33–36) yielded clinically meaningful levels of secreted FVIII activity without triggering cellular stress or altering PLCs’ inherently immune-inert state (22).

These prior studies with PLCs, and studies performed in other promising cell types, have collectively provided compelling evidence that efficient gene addition and long-term FVIII expression can be achieved following transduction with integrating vectors, such as those based upon murine retroviruses or lentiviruses (31, 34, 37–48). While we found no evidence of integration within or near oncogenes or tumor repressors in the lentiviral vector-transduced PLCs, we undertook the present studies to explore whether we could identify an even safer gene delivery approach, testing the hypothesis that using CRISPR/Cas9 to achieve site-specific insertion of an optimized fVIII transgene (lcoET3 (22, 22, 36, 49)) cassette into a genomic “safe harbor” would allow efficient gene addition, while avoiding the potential for insertional mutagenesis that is inherent to all integrating viral vectors (50–54). The specific genomic site we selected for this initial proof-of-concept was the AAVS1 site on chromosome 19, as integration into this site does not disrupt any essential genes, yet the site is transcriptionally active, ensuring inserted transgene cassettes are stably and robustly expressed (55). As liver sinusoid-derived endothelial cells (LSECs) are the natural producers of FVIII in the body (56) and are thus being explored as cellular platforms to treat HA (57), we also performed studies to ascertain whether it is possible to use CRISPR/Cas9 to efficiently mediate delivery of a fVIII transgene cassette into the AAVS1 locus in human (h)LSECs. With each cell type, we compare the success and efficiency of the CRISPR/Cas9-based approach to that achieved when the same fVIII transgene cassette was delivered *via* a lentiviral vector (LV-lcoET3) or transfection with a plasmid (Plasmid-lcoET3). We report that human PLCs and LSECs can both be efficiently transduced with lentiviral vectors encoding a bioengineered lcoET3 fVIII transgene and subsequently produce and secrete therapeutically relevant levels of FVIII procoagulant activity. Surprisingly, however, we found that both cell types appear to be largely refractory to CRISPR/Cas9-mediated knockin of the lcoET3 fVIII transgene in the AAVS1 genome locus. Nevertheless, the ability to successfully insert an RFP reporter into this locus using an identical procedure and donor template plasmid suggest the failure to achieve knockin of lcoET3 at this site is likely a function of the large size of the lcoET3 expression cassette. Interestingly, despite its inability to mediate integration of the lcoET3 cassette at the AAVS1 locus, the use of plasmid-based transfection to deliver the CRISPR/Cas9-based gene editing machinery to PLCs still upregulated expression of toll-like receptor (TLR) molecules TLR-3 and TLR-7, indicating that such manipulation may compromise the immune-inert properties that make these cells desirable as an off-the-shelf product, and highlighting the importance of selecting the appropriate delivery system if CRISPR/Cas9-edited human cells are ultimately to be used clinically.

## Materials and methods

### Isolation and culture of PLCs and hLSECs

Human placenta-derived mesenchymal stromal cells (PLCs) were isolated as previously described (22). In brief, as per the guidelines from the Office of Human Research Protection at Wake Forest University Health Sciences, human placentas were acquired from full-term deliveries. To isolate the placental stromal/stem cells, the tissue was minced, and enzymatic digestion was carried out, followed by culture in a 37°C humidified incubator with 5% CO_2_ in placental cell growth media (PCGM), which consisted of α-minimum essential medium (α-MEM) supplemented with 15% fetal bovine serum (FBS), 19% AmnioMAX, 1% GlutaMAX, and 2.5 μg/mL gentamicin (Thermo Fisher Scientific, Waltham, MA). After 2-3 weeks of culture upon reaching 70-80% confluency, PLCs were passaged with TrypLE (Thermo Fisher Scientific, Waltham, MA) and positively selected for CD117/c-kit using c-kit selection microbeads (Miltenyi Biotec, Auburn, CA, USA) as per manufacturer’s protocol. After c-kit selection, cells were seeded at a density of 3000-4000 cells/cm^2^ and expanded in PCGM. Primary human liver sinusoid-derived endothelial cells (hLSECs) were purchased at Passage 2 from Lonza and cultured in endothelial growth medium 2 (EGM-2; Lonza, Walkersville, MD) using culture flasks coated with rat tail collagen 1 (Corning, Corning, NY) in a 37°C incubator with 5% CO_2_.

### Plasmid construction and synthesis

An AAVS1 transgene knock-in vector kit, consisting of the pCas-Guide-AAVS1 (GE100023) and pAAVS1-puro-DNR (GE100024) plasmids, was purchased from OriGene Technologies, Inc. (Rockville, MD). A “cassette” containing the bioengineered fVIII transgene “lcoET3” under the transcriptional control of the constitutively active human EF1α promoter was cloned into the multiple cloning site (MCS) of the pAAVS1-puro-DNR plasmid using standard restriction enzyme digestion and ligation protocols and reagents (New England Biolabs/NEB, Ipswich, MA). lcoET3 is a liver-codon-optimized chimeric human/porcine fVIII transgene containing a designed to produce bioengineered fVIII with increased FVIII secretion efficiency and activity (22, 36). Both plasmids contain the ampicillin resistance gene. Therefore, they were transfected into competent *E. coli* cells (NEB) using heat shock, and the transformants were plated on LB agar plates containing ampicillin (100 μg/mL). Ampicillin-resistant colonies were plucked 24 hours later and inoculated into LB broth. Once the cultures were expanded, the plasmids from the *E. coli* were isolated using the Plasmid *Plus* Midi kit (Qiagen, Valencia, CA), and restriction enzyme digestion was performed followed by 2% agarose gel electrophoresis to verify the identity and integrity of the plasmid.

### CRISPR/Cas9-mediated knock in of fVIII transgene and transfection with lcoET3 plasmid

To carry out CRISPR/Cas9-mediated gene addition of the EF1α-lcoET3 expression cassette into the AAVS1 locus, we utilized the AAVS1 Transgene Knockin kit (OriGene, Cat# GE100027), following the manufacturer’s instructions (OriGene Technologies, Inc., Rockville, MD). In brief, PvuI/SpeI were used to cut the EF1α-lcoET3 expression cassette from the pLenti-EF1a-lcoET3 lentivector backbone plasmid. The excised cassette was then inserted into the pAAVS1-Puro-DNR donor template plasmid (OriGene, Cat# GE100024) using CloneEZ^®^ PCR Cloning (Genscript USA, Inc., Piscataway, NJ) to directly insert the modified fragment into the right position while removing the existing CMV promoter. This entire custom cloning procedure was performed by GenScript USA, Inc. To achieve CRISPR/Cas9-mediated insertion of the EF1α-lcoET3 cassette at the AAVS1 site, hLSECs and PLCs were co-transfected with the pAAVS1-EF1α-lcoET3-Puro-DNR donor template plasmid (carrying the EF1α-lcoET3 expression cassette flanked by homology arms to the AAVS1 locus) and the pCas-Guide-AAVS1 plasmid encoding both the sgRNA to the AAVS1 site and Cas9 (OriGene Cat# GE100023) using Turbofectin 8.0 Transfection Reagent, following the manufacturer’s instructions (OriGene Technologies, Inc.). Briefly, an appropriate number of PLCs/hLSECs were plated to achieve 50-70% confluency the next day. The following reagents were added in the specified order, mixing as indicated, to 250 μL of Opti-MEM I (Thermo Fisher Scientific, Waltham, MA) to prepare the transfection medium: 1 μg of pCas-Guide-AAVS1, vortex gently; 1 μg of pAAVS1-EF1α-lcoET3-Puro-DNR, vortex gently; and 6 μL of Turbofectin 8.0 (OriGene Technologies, Inc.), pipette gently to mix. The mixture was incubated at room temperature for 15 minutes. The media in the culture was replaced with fresh media, and the transfection medium was added dropwise. The culture flask was gently rocked back-and-forth to distribute the complex and was returned to the 37°C incubator with 5% C02. The cells were cultured for 3 weeks, passing as needed, to dilute out cells with the plasmids in episomal form. PLCs were then selected using puromycin (Gibco, Amarillo, TX). The appropriate puromycin concentration was determined based on a kill curve (0.4 μg/mL for PLCs). A similar procedure was carried out using the pAAVS1-EF1α-lcoET3-Puro-DNR plasmid alone, as a control for gene delivery efficiency arising from transient transfection alone.

### Transduction with lentiviral vector encoding lcoET3

Cells were plated to reach 50-60% confluency the following day. Once confirming by microscopy that the cells were healthy, the media was removed and the cells were washed with QBSF-60 (Quality Biologicals, Gaithersburg, MD, USA). This was followed by transduction in QBSF-60 containing 8 μg/mL protamine sulfate (Fresenius Kabi, Lake Zurich, IL) and the third generation self-inactivating lentiviral vector encoding the lcoET3fVIII transgene under the transcriptional control of the constitutive human EF-1α promoter at an MOI (multiplicity of infection) of 7 (additional MOI of 7 after 4 h). After transduction, cells were washed and refed with fresh complete media and passaged three times before analyses were performed.

### Determination of vector copy number

The provirus copy number per diploid human genome in lentivector-transduced (LV-lcoET3) cells was determined using the Lenti-X Provirus Quantitation Kit (Takara, Mountain View, CA) according to the manufacturer’s protocol. Briefly, genomic DNA was isolated from transduced and non-transduced cells using the NucleoSpin Tissue kit (Takara, Mountain View, CA). Serial dilutions were made with genomic DNA, and qPCR amplification was carried out along with a standard curve derived from serial dilutions of calibrated provirus control template. The raw Ct values of the sample were correlated to the standard curve to determine the provirus copy number per cell. A correction coefficient was also incorporated to compensate for the different PCR sensitivities to amplifying provirus control template only vs. provirus sequence integrated in genomic DNA.

### Flow cytometric analysis of cells for TLRs and ER stress molecules

Flow cytometric analysis of PLCs and hLSECs was performed using antibodies for TLR 3,4,7,8,9 (ab45093, ab11227, ab45097, ab58864, Abcam, Cambridge, UK [TLR 3,4,8,9] and IC5875P, R&D Systems [TLR 7]) and BiP/CHOP (Cell Signaling Technology, Danvers, MA). Intracellular staining was performed as per manufacturer’s protocol using the Intracellular Flow Cytometry kit (Cell Signaling Technology, Danvers, MA) for TLR 3,7,8,9 and BiP/CHOP. Cells were directly stained and fixed for TLR 4. After processing, cells were analyzed using a BD Accuri C6 flow cytometer, and the data were analyzed using FlowJo Software (BD Biosciences, San Jose, CA). Cells treated with tunicamycin (1 μg/mL overnight for BiP and 2 μg/mL 8 h for CHOP) were used as positive control for ER stress.

### Endpoint PCR to detect bioengineered fVIII in gene-modified cells

To determine whether the transgene was present in the gene-modified cells, genomic DNA was isolated post puromycin selection using the DNeasy Mini kit (Qiagen, Valencia, CA). The PCR reaction was carried out in a volume of 50 μL using 200 ng gDNA and primers specific for the lcoET3 fVIII transgene (forward: 5’-TTTCCGTCCTCAGCCGTCGC-3’ and reverse: 5’-AGGACAGCTCCACAGCTCCCA-3’). gDNA from unmodified cells was used as a negative control in addition to a no-template water control. The cycle conditions were as follows: 1 cycle of initial denaturation at 94°C for 3 min, followed by 35 cycles of 94°C for 18 s, 60°C for 18 s, and 72°C for 30 s, and final extension at 72°C for 10 min (Platinum Blue Supermix, Invitrogen, Waltham, MA). Agarose (1%) gel electrophoresis was then carried out to visualize the PCR products.

### RT-qPCR for assessing FVIII, RFP, and PPP1R12C mRNA expression

RNA was extracted from the cells using RNeasy Mini kit (Qiagen, Valencia, CA, USA). The concentration of RNA was measured using NanoDrop 2000 (Thermo Fisher Scientific, Wilmington, DE, USA), and the integrity of RNA was verified using RNA 6000 Nano kit and 2100 Bioanalyzer (Agilent, Santa Clara, CA, USA). gDNA contamination was eliminated using RQ1 RNase-free DNase (Promega, Madison, WI, USA), and RNA was converted to cDNA using the Omniscript RT kit (Qiagen, Valencia, CA, USA). RT-qPCR was then carried out to compare the expression of endogenous FVIII, exogenous lcoET3, and RFP between unmodified control, LV-lcoET3, plasmid-lcoET3, and CRISPR-lcoET3 cells. RT-qPCR was also performed to compare the expression of *PPP1R12C, the gene encoded by the AAVS1 locus, in PLCs, hLSECs, and human iPSC to assess the accessibility of this region to CRISPR/Cas9-mediated editing in each cell type.* The following sets of gene-specific primers were used for these studies:

lcoET3 transgene:
forward: 5’CTGGGCCCATCTGTGCTGTA-3’
reverse 5’-TCCAAGGTGGTCTTGGCCTG-3’

human FVIII B domain (lcoET3 is B domain-deleted):
forward 5’-TCTCCCGAAACCAGACTTGC-3’
reverse: 5’-GTTCCCTGAAGAAGGCTCCC-3’)

human GAPDH (as the housekeeping gene for normalization):
forward 5’-CACTGCTGGGGAGTCCCTGC-3’
reverse 5’-GCACAGGGTACTTTATTGATGG-3’

RFP primer set #1 (Amplicon size = 92 bp):
forward 5’- TCC GAG GGC GAA GGC AAG -3’
reverse 5’- AGG ATG TCG AAG GCG AAG GG -3’

RFP primer set #2 (Amplicon size = 139 bp):
forward 5’- TCA TGT ACG GCA GCA AAG CC -3’
reverse 5’- GTG TCC TGG GTA GCG GTC A -3’

*PPP1R12C* :
forward 5’-GGCCTGCATTGATGAGAA-3’
reverse 5’-GAGGTACCTGGCGATATCTA-3’

The SYBR green–based TB Green Advantage Mastermix (Takara, Mountain View, CA), along with the primers (200 nM) and cDNA template (100 ng), was added to a MicroAmp Optical 96-well Reaction plate (Thermo Fisher Scientific, Waltham, MA). The PCR reaction was performed using the following settings: 1 cycle of initial denaturation at 95°C for 15 s, followed by 40 cycles of 95°C for 4 s, [60°C for 15 s and 72°C for 10 s, for lcoET3 and hFVIII] or [60°C for 1 minute, for *PPP1R12C* and RFP], and standard melt curve analysis, and the amplification was read using the Quant Studio 3 real-time PCR (Applied Biosystems, Waltham, MA).

### Immunofluorescence microscopy for visualizing intracellular FVIII

Cells were grown on chamber slides (Thermo Fisher Scientific, Waltham, MA) to reach 60-70% confluency, and were then fixed with 4% paraformaldehyde. Immunofluorescence staining for FVIII was performed by permeabilization with 0.2% Triton-X for 15 min, blocking with protein block (Dako, Santa Clara, CA) for 30 min at RT, and incubation at 4°C overnight with a mouse monoclonal anti-human FVIII primary antibody (ESH-8, Sekisui Diagnostics, Burlington, MA) at a dilution of 1:150. The slides were washed the following day and incubated with a goat anti-Mouse AlexaFluor^®^ 594-conjugated secondary antibody (ab150116, Abcam, Cambridge, UK) at a dilution of 1:500. Finally, the nuclei were counterstained with DAPI (1:1000) and coverslips were mounted with ProLong Gold Antifade Mounting Medium (Thermo Fisher Scientific), followed by sealing with clear nail polish. The slides were imaged using either a Leica DM4000B or an Olympus BX63 fluorescence microscope. A control slide, stained with the secondary antibody only, was included to assess the degree of non-specific binding and determine the appropriate exposure settings for image acquisition.

### Immunofluorescence microscopy for visualizing intracellular RFP

Cells (unmodified, transfected with pAAVS1-(CMV)-RFP-Puro-DNR, or edited with CRISPR/Cas9 to insert the CMV-RFP expression cassette into the AAVS1 locus) were grown on chamber slides (Thermo Fisher Scientific, Waltham, MA) to reach 60-70% confluency, and were then fixed with 4% paraformaldehyde. Immunofluorescence staining for RFP was performed by permeabilization with 0.2% Triton-X for 15 min, blocking with protein block (Dako, Santa Clara, CA) for 30 min at RT, and incubation at 4°C overnight with a rabbit polyclonal anti-RFP primary antibody (ab167453, Abcam, Cambridge, UK) at a dilution of 1:500. The slides were washed the following day and incubated with a goat anti-Rabbit AlexaFluor^®^ 594-conjugated secondary antibody (A-11072, Thermo Fisher Scientific) at a dilution of 1:500. Finally, the nuclei were counterstained with DAPI (1:1000) and coverslips were mounted with ProLong Gold Antifade Mounting Medium (Thermo Fisher Scientific), followed by sealing with clear nail polish. The slides were imaged using a Leica DM4000B fluorescence microscope. A control slide, stained with the secondary antibody only, was included to assess the degree of non-specific binding and determine the appropriate exposure settings for image acquisition.

### aPTT assay to quantify clotting activity of FVIII secreted into cell supernatants

Cells were plated to reach 30-40% confluency the next day. Media was replaced with phenol red-free PCGM/EGM-2 the following day. After 24 h of incubation, the media was collected, centrifuged to remove debris, aliquoted (after proper mixing), and stored at -80°C until they were sent out to perform aPTT (activated partial thromboplastin time) assays. These assays were performed by the Wake Forest Baptist Medical Center Special Hematology Laboratory according to standard clinical procedures, using a Top 300 CTS clinical coagulometer (Instrumentation Laboratories, Bedford, MA, USA). Cell counts were also performed and correlated with the aPTT results, enabling data to be presented as IU/24h/10^6^cells.

### WGS for assessing integration of lcoET3 fVIII transgene in the AAVS1 locus

Genomic DNA collected from cells was subjected to 2% agarose gel electrophoresis to verify integrity, and concentration was measured using the Qubit 3.0 Fluorometer (Thermo Fisher Scientific, Waltham, MA). Whole genome sequencing (WGS) was carried out with 40X coverage on DNBSEQ™ NGS technology platform by BGI Genomics (Shenzhen, Guangdong, China). The standard NCBI human reference genome GRCh38 (chromosome 19) was downloaded, and the 9 kb CRISPR sequence was inserted using AWK. The reference genome containing the intended insertion sequence was then used to map (FASTQC) the reads from WGS to ascertain whether the lcoET3 expression cassette had been successfully inserted into the AAVS1 locus.

### Impact of gene delivery on cell viability

To assess the impact of each gene delivery platform/method on the viability of PLCs and hLSECs, viability of each cell population (n=3) was quantified at various time post-gene delivery using a NucleoCounter^®^ NC-200™ (ChemoMetec, Denmark) or a Countess™ 3 (Invitrogen, Waltham, MA), following the protocols provided by the respective manufacturer.

### Statistical analysis

All experimental results are presented as mean +/- the standard error of mean (SEM) with the number of replicates (n) indicated. GraphPad Prism 9 was used to perform all statistical analyses (one-way ANOVA followed by *post hoc* Tukey test), and p-value < 0.05 was considered to be statistically significant.

## Results

**Figure 1:**
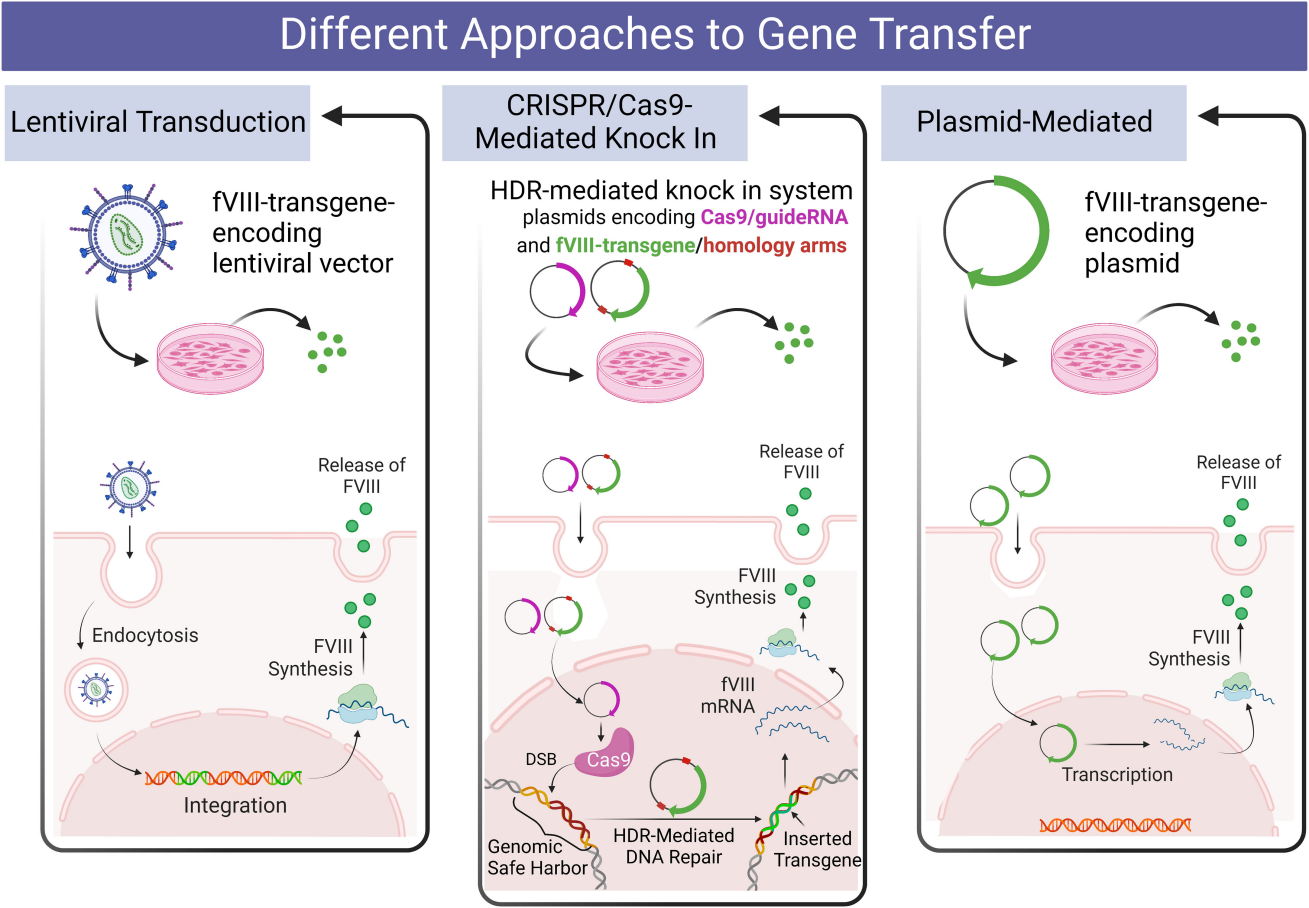
Diagrammatic overview of the design of the present studies comparing various methods of delivering a fVIII transgene to human PLCs and hLSECs.

A diagrammatic overview of the various gene delivery approaches tested in hese studies appears in **Figure 1**.

### CRISPR/Cas9 engineering using a dual-plasmid system

lcoET3 is a liver-codon-optimized (lco) bioengineered transgene that contains high-expression elements of porcine *F8* (ET3) (36). To achieve insertion of the lcoET3 transgene into the AAVS1 locus, PLCs (n = 5) and hLSECs (n = 3) were transfected with a dual-plasmid system (**Figure S1**) containing a Cas9/AAVS1 guide RNA-expressing plasmid and a donor plasmid into which the EF1α-lcoET3 expression cassette was commercially cloned (GenScript), between the left and right AAVS1 homologous “arm” sequences. The success of cloning was verified using restriction enzyme digestion (**Figure S2**).

### Endpoint PCR confirms the presence of the lcoET3 transgene in gene-modified cells

**Figure 2:**
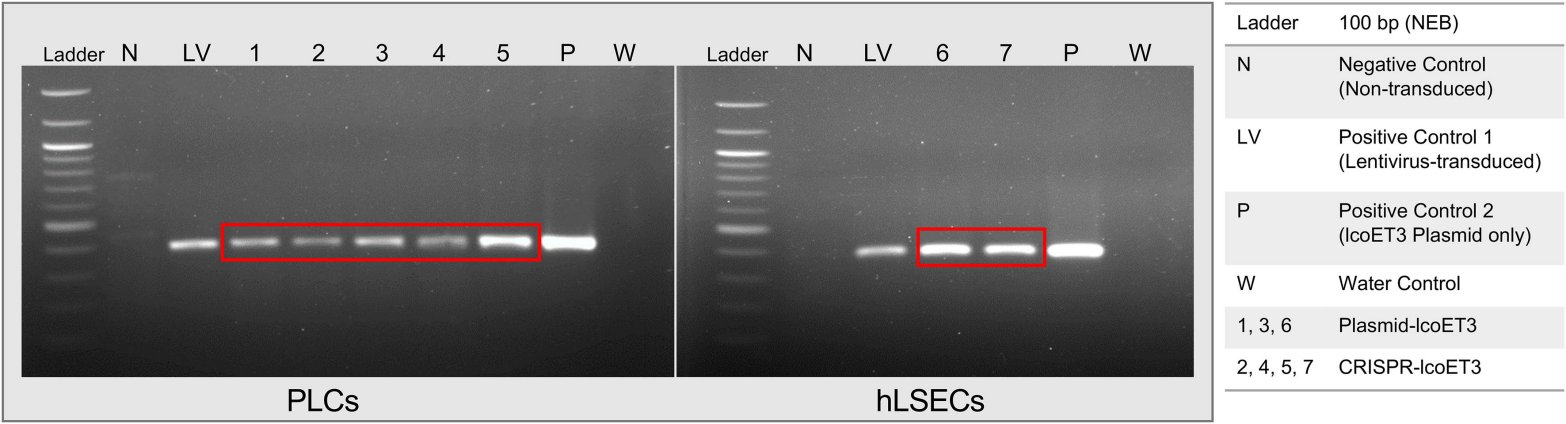
Verification of lcoET3 transgene DNA in gene-modified PLCs and hLSECs using PCR. DNA was extracted from control and gene-modified cells (3 passages after transduction/transfection) and subjected to PCR with primers designed to amplify a 395-bp region within the lcoET3 sequence. Agarose gel electrophoresis was then performed to visualize the size of the PCR products. Unmodified control cells and a reaction containing all constituents of the PCR except for template DNA (water/no-template) were used as negative controls while LV-lcoET3 cells and pure lcoET3 plasmid were used as positive controls.

Endpoint PCR using primers specific for lcoET3 was performed with genomic DNA isolated from gene-modified cells (LV-lcoET3, CRISPR-lcoET3, and Plasmid-lcoET3) and control (unmodified) cells that were passaged a minimum of 3 times prior to DNA isolation. The PCR products were run on a 1% agarose gel with a 100 bp ladder (New England Biolabs, Ipswich, MA). The lcoET3-specific primers yield a 395 bp amplicon. **Figure 2** shows the correct amplicon is present in all gene-modified cells (PLCs: *n*=5, hLSECs: *n*=3), while no product was observed in the unmodified control (N) or the no-template/water control (W).

### RT-qPCR detection of lcoET3 expression in gene-modified cells

**Figure 3:**
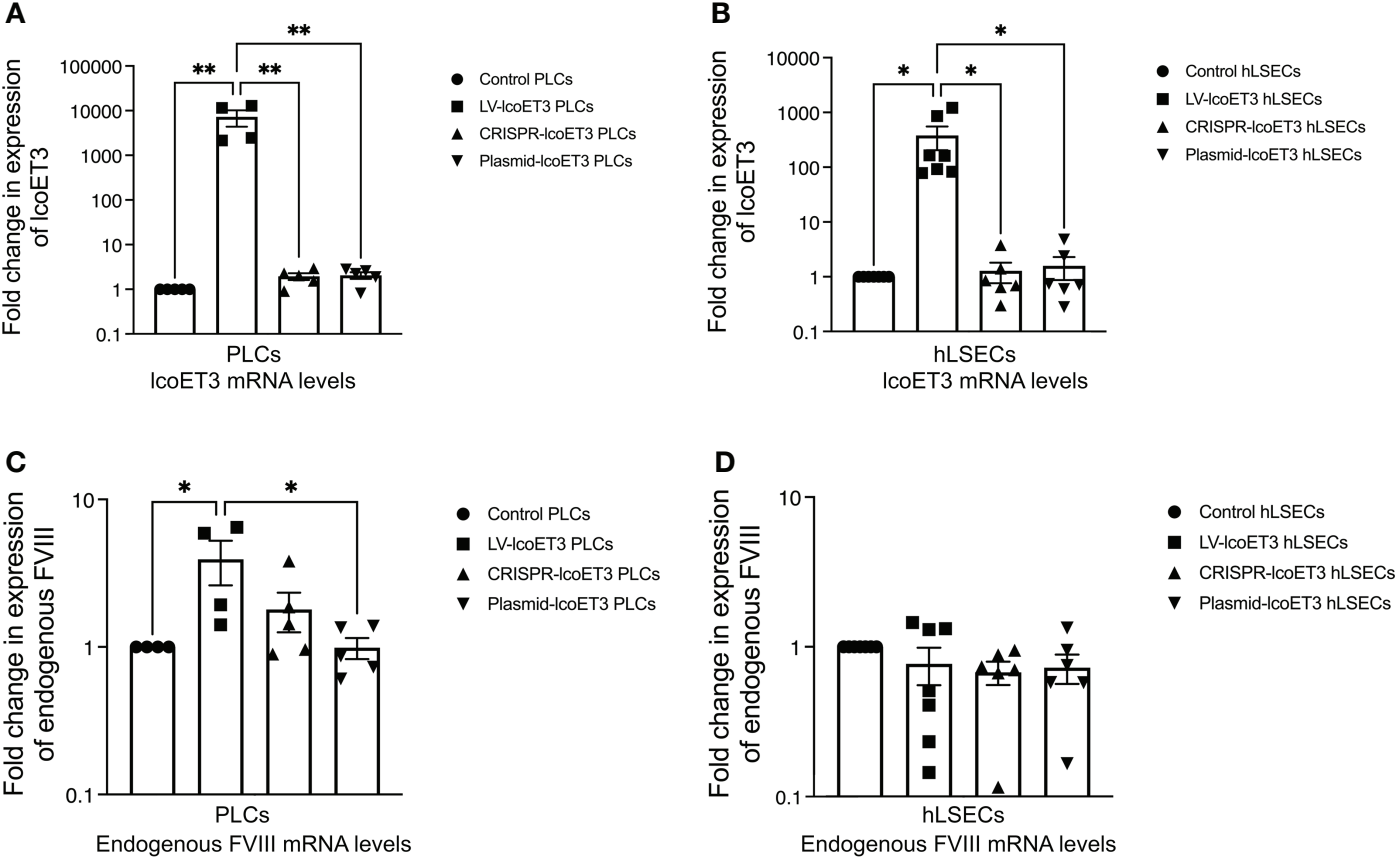
Detection of FVIII mRNA levels by RT-qPCR. Relative fold-change in expression of lcoET3 transgene **(A, B)** and endogenous FVIII mRNA **(C, D)** in gene-modified PLCs **(A, C)** and hLSECs **(B, D)** is presented in comparison to unmodified control after normalization to amplification of GAPDH in each respective sample. n = 5 for unmodified/control PLCs, n=4 for LV-lcoET3 PLCs, n = 5 for CRISPR-edited PLCs, n=5 for Plasmid-lcoET3-transfected PLCs; n = 6-8 for all hLSEC treatment groups. *p<0.05; **p<0.01.

In order to investigate whether the gene-modified PLCs and hLSECs were expressing lcoET3, the levels of the mRNAs lcoET3 and human GAPDH were quantified in CRISPR-modified, lentivector-transduced, plasmid-transfected, and unmodified cells (passaged a minimum of 3 times prior to RNA isolation) by RT-qPCR using the appropriate transcript-specific primers. The raw Ct values were normalized to GAPDH to obtain a ΔCt value, which was then compared with the negative control (unmodified cells) to obtain each sample’s DΔCt value. The formula 2^-ΔΔCt^ was then used to represent the fold-change in expression of the different genes. All gene-modified PLCs (N=3 biological replicates; technical replicates: CRISPR-edited: *n*=5; lentivector-transduced: *n*=3; plasmid transfected: *n*=3) and hLSECs (N=3 biological replicates; technical replicates: CRISPR-edited: *n*=3; lentivector-transduced: *n*=3; plasmid transfected: *n*=3) exhibited detectable levels of lcoET3 mRNA that exceeded the minimal background amplification seen in the unmodified control group (*n*=4) in 4 independent experiments. However, only lentiviral transduction yielded levels of lcoET3 mRNA expression that were statistically elevated above the background seen in control unmodified PLCs and hLSECs. Indeed, the levels of lcoET3 mRNA expression achieved following lentivector transduction were quite robust, being ~5000-times and 800-times that seen in the CRISPR/Cas9-edited PLCs and hLSECs, respectively (**Figures 3A, B**).

To assess whether lcoET3 expression affected steady-state endogenous FVIII expression in PLCs and/or hLSECs, FVIII mRNA levels were compared between unmodified control and gene-modified cells (**Figures 3C, D**). No significant change in endogenous FVIII expression was observed after gene modification in either cell type, demonstrating that forced expression of a bioengineered fVIII transgene does not impact expression from the endogenous FVIII locus.

### Evaluation of FVIII production and activity

**Figure 4:**
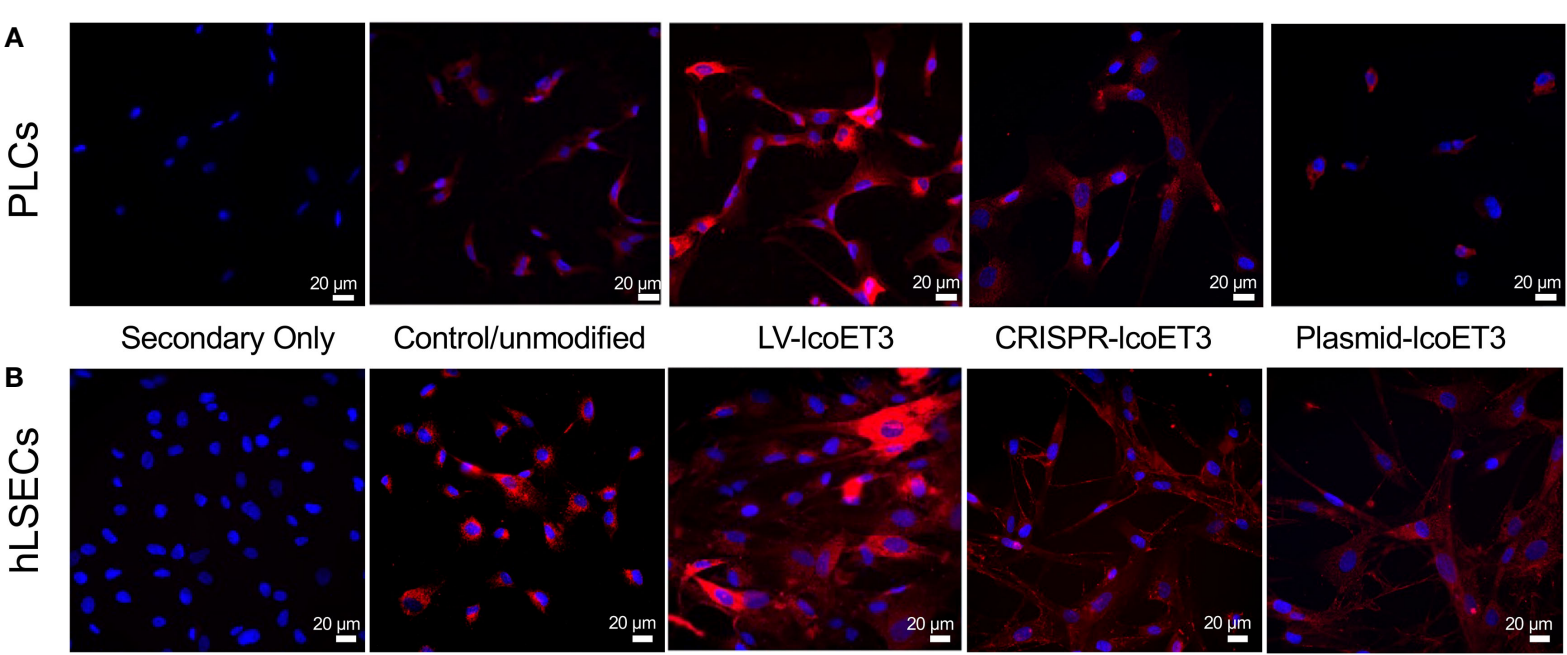
Representative images of immunofluorescence analysis of intracellular FVIII protein in unmodified and gene-modified PLCs and hLSECs. PLCs **(A)** and hLSECs **(B)** in each treatment group were stained with a primary antibody specific for FVIII that was then detected with an AlexaFluor^®^ 594-conjugated secondary antibody (red) and nuclei were counterstained with DAPI (blue). Controls consisted of slides with unmodified PLCs and hLSECs stained identically and slides of each cell type stained with secondary antibody alone to establish levels of background fluorescence.

**Figure 5:**
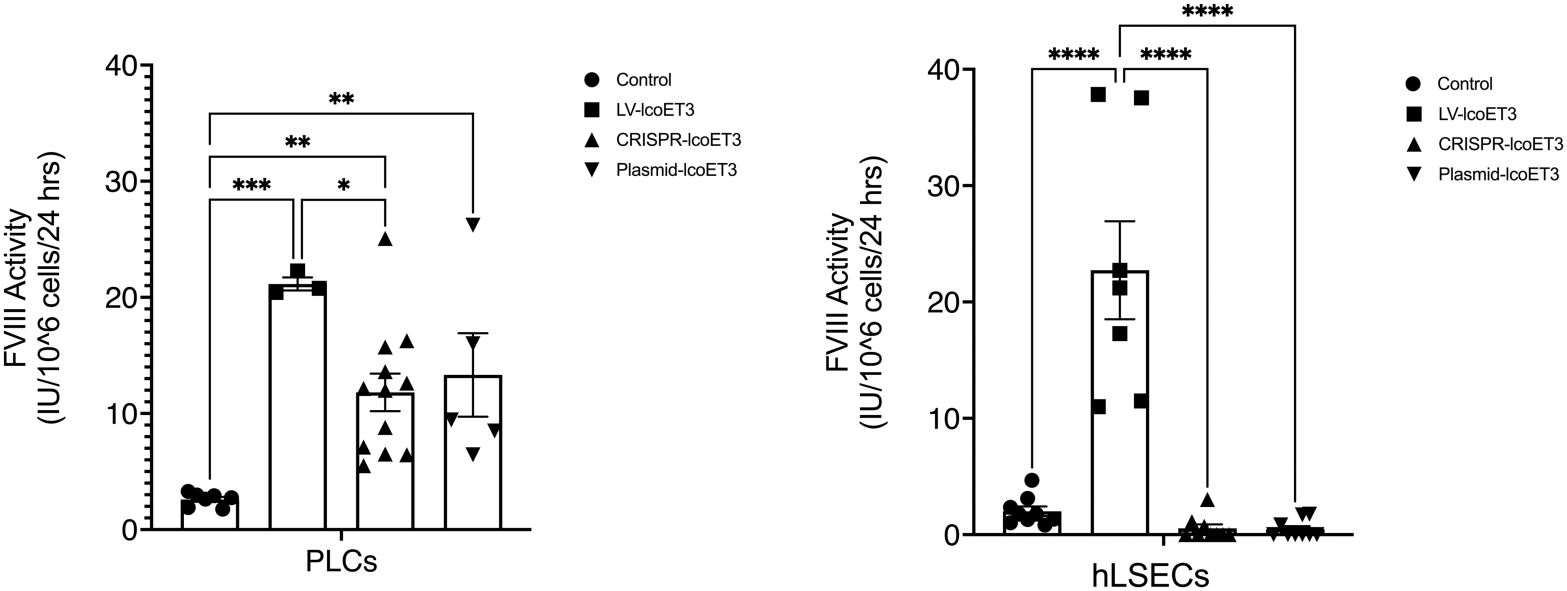
Evaluation of FVIII coagulation activity by activated partial thromboplastin time (aPTT) assay performed on 24h culture supernatants after normalization for cell number in each sample. Data are presented as the amount of functional FVIII in International Units (IU) being produced by 10^6^ cells in 24h. (**A** – PLCs, **B** – hLSECs). n = 7 for unmodified/control PLCs, n = 3 for LV-lcoET3 PLCs, n = 12 for CRISPR-edited PLCs, and n = 5 for Plasmid-lcoET3-transfected PLCs; n = 7-9 for all hLSEC groups. *p<0.05; **p<0.01; ***p<0.001; ****p<0.0001.

After verifying the expression of lcoET3 FVIII mRNA, immunofluorescence microscopy was used to determine the presence and levels of lcoET3/FVIII protein in the gene-modified PLCs (**Figure 4A**) and hLSECs (**Figure 4B**). As can be seen in these figures, intracellular lcoET3/FVIII protein is readily detected in all groups compared to the negative control (secondary antibody alone), including the unmodified PLCs and hLSECs, both of which constitutively express endogenous FVIII. As was seen at the RNA level, no significant change was seen in the levels of lcoET3/FVIII protein in the CRISPR-edited or plasmid-transfected PLCs or hLSECs, but the lentivector-transduced group (LV-lcoET3) of both PLCs and hLSECs exhibited markedly enhanced levels of lcoET3/FVIII protein. The functionality of the secreted FVIII protein was then measured by performing an activated partial thromboplastin time (aPTT)-based one-stage coagulation assay on cell culture supernatants harvested from cells over the course of 24 h. The coagulation time in this assay is then translated into units of FVIII activity to determine the potential for these cells to correct the disease phenotype. The raw data from the aPTT assay was normalized to the volume of media collected and the cell number present in each sample to yield the final result presented as FVIII IU/10^6^ cells/24 h. A significant increase (p>0.05) in FVIII activity was only observed in the lentivector-transduced (LV-lcoET3) groups of both cell types (N=3 biological replicates for PLCs and N=2 biological replicates for hLSECs; n=3 technical replicates for PLCs and n=9 technical replicates for hLSECs). In agreement with the RT-qPCR and immunofluorescence data, neither the PLCs (N=5 biological replicates) nor the hLSECs (N=3 biological replicates) that were gene-edited with CRISPR/Cas9 (n=12 technical replicates for PLCs and n=9 for hLSECs) or transfected with the lcoET3 plasmid (n=5 technical replicates for PLCs and n=9 for hLSECs) exhibited a statistically significant increase in FVIII activity over that of unmodified cells (N=3 biological replicates and n=5 technical replicates for PLCs; N=3 biological replicates and n=9 for hLSECs) (**Figure 5**).

### Assessment of whether gene delivery alters viability of PLCs or hLSECs

We next investigated whether gene modification, *via* plasmid transfection, lentivector transduction, or CRISPR/Cas9-mediated editing, PLCs and hLSECs subjected to each gene modification (and unmodified as controls/reference) were analyzed using a NucleoCounter^®^ NC-200™ or a Countess™ 3. No significant difference in viability was observed post gene transfer, irrespective of the method used for gene transfer (data not shown).

### Assessment of whether gene delivery dysregulates innate immunity and/or stress molecules

**Figure 6:**
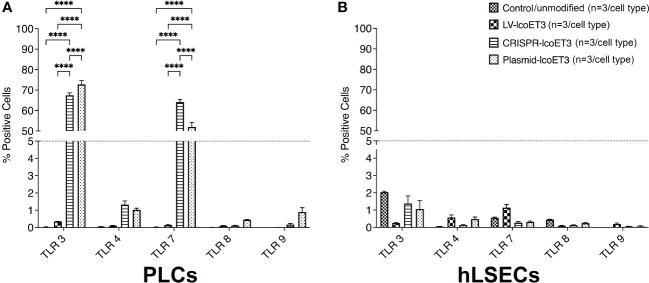
Flow cytometric analysis to quantify expression of Toll-like receptors (TLRs) on unmodified and gene-modified PLCs **(A)** and hLSECs **(B)**. n = 3/experimental group for each cell type. ****p<0.0001.

We next investigated whether gene modification induced up-regulation of toll-like receptors (TLR), key molecules in the innate immune response to foreign genetic material (58–60). Flow cytometric analysis demonstrated that unmodified PLCs did not express detectable levels of TLR 3, 4, 7, 8, or 9. However, transfection of PLCs with either the lcoET3-expressing plasmid alone (Plasmid-lcoET3) or with the Cas9 + lcoET3-expressing plasmids (CRISPR-lcoET3) resulted in robust up-regulation of TLR 3 and TLR 7 in PLCs, such that > 65% of plasmid-transfected PLCs expressed TLR 3 and > 50% of plasmid-transfected PLCs expressed TLR 7 (**Figure 6**). Interestingly, transfection with the same plasmids did not elicit upregulation in any of the TLRs in hLSECs. In agreement with what we reported in prior studies with bone marrow-derived mesenchymal stromal cells (61), transduction of PLCs and hLSECs with lentiviral vectors did not induce expression of TLR molecules on a significant percentage of the modified cells (< 2%).

**Figure 7:**
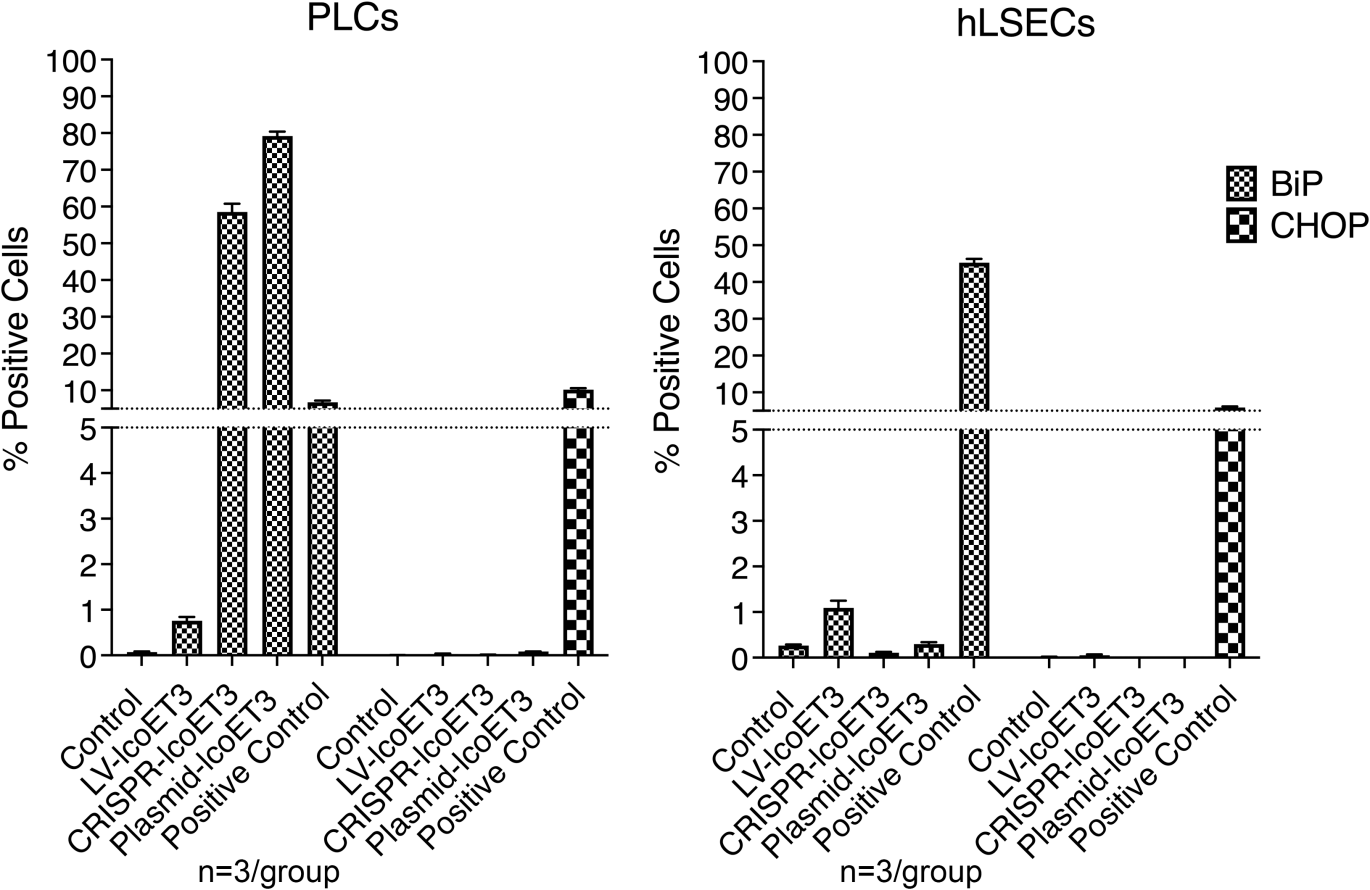
Flow cytometric analysis to quantify expression of ER stress molecules BiP and CHOP in unmodified and gene-modified PLCs (left panel) and hLSECs (right panel). Cells treated with tunicamycin were used as positive controls for ER stress. n=3/experimental group for each cell type.

Since FVIII is a relatively large protein and can place significant stress on the endoplasmic reticulum (ER) and the downstream secretory pathway, we used flow cytometry to investigate whether there was an up-regulation in the unfolded protein response (UPR) sentinel chaperone BiP (binding immunoglobulin protein) and downstream signaling proapoptotic protein C/EBP homologous protein (CHOP) (17, 33, 62, 63). Tunicamycin-treated cells were used as positive controls. Compared to the unmodified control cells, the only groups that exhibited a significantly increased percentage of cells expressing BiP were the positive controls and the PLCs that were transfected with plasmids (CRISPR-lcoET3 and Plasmid-lcoET3) (**Figure 7**). With respect to CHOP, only the positive control group showed a significantly increased percentage of cells expressing this stress molecule. The percentages of cells expressing both BiP and CHOP were very low (<2%) in all other groups.

### Whole genome sequencing to assess integration at AAVS1 site

**Figure 8:**
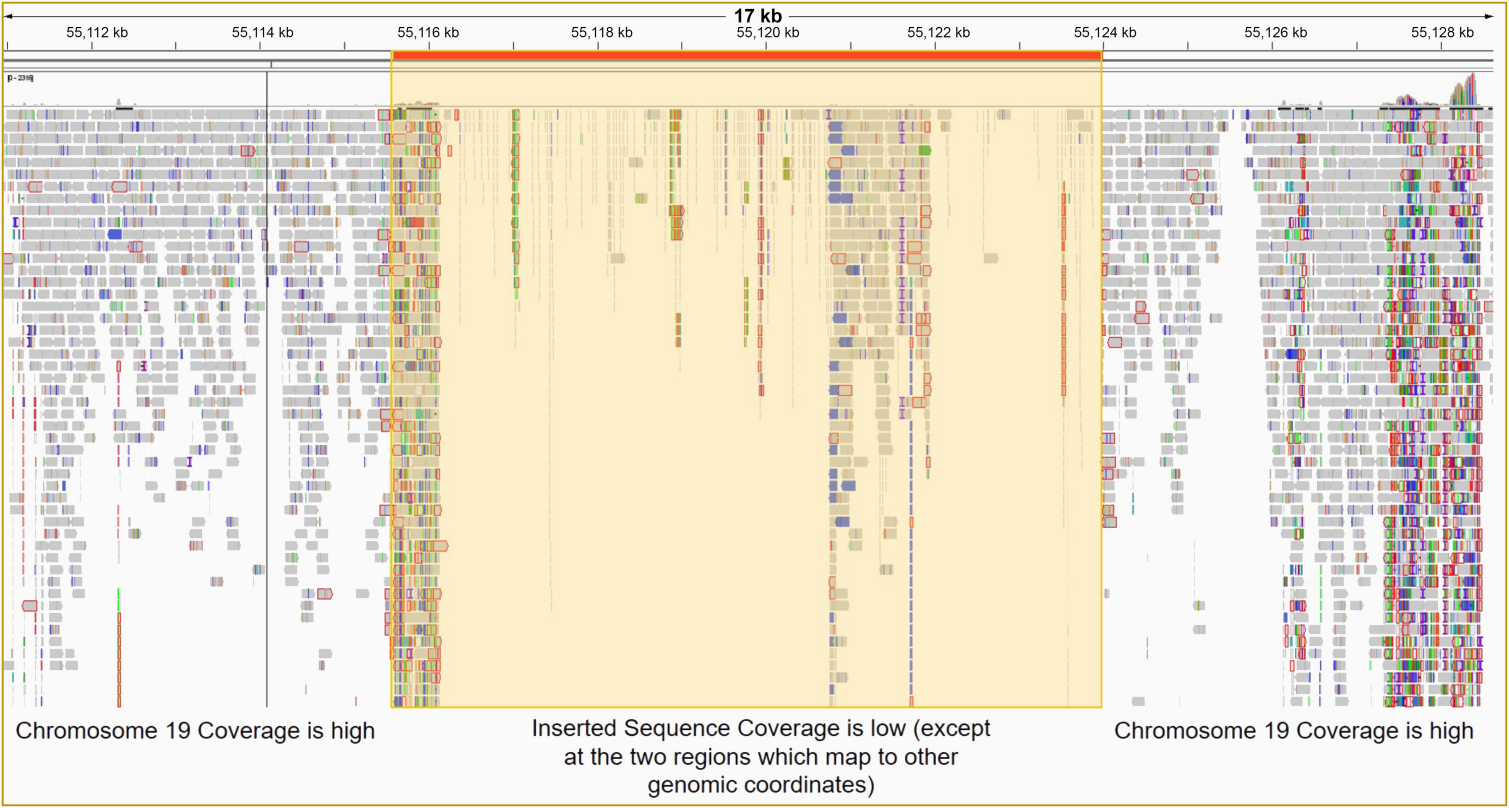
Whole genome sequencing (WGS) of PLCs to determine whether CRISPR/Cas9 editing led to successful insertion of the EF1α-lcoET3 expression cassette at the AAVS1 genomic locus. After ensuring DNA integrity, WGS was performed with 40X coverage using the DNBSEQ™ NGS platform, and the sequence reads were aligned to a standard reference human genome containing the inserted sequence at the desired locus.

Given that the RT-qPCR, immunofluorescence, and aPTT data did not suggest we had successfully inserted the EF1α-lcoET3 expression cassette into the AAVS1 genomic locus, yet the endpoint PCR demonstrated the presence of DNA for the lcoET3 transgene within the CRISPR-edited PLCs that had been selected in puromycin, we next performed whole genome sequencing (WGS) on DNA from the putatively CRISPR-edited PLCs at 40x coverage and aligned the reads to a reference genome that had been modified *in silico* to contain the putative insertion at the AAVS1 site. As can be seen in **Figure 8**, these analyses did not demonstrate any significant coverage/alignments in this region, thus providing sequence-level proof for the apparent failure to successfully achieve CRISPR-mediated insertion of the EF1α-lcoET3 cassette at the AAVS1 locus in the PLCs.

### Evaluation of whether the inaccessibility of the AAVS 1 locus and/or the size of the lcoET3 expression cassette are factors limiting CRISPR/Cas9-mediated gene editing in PLCS and hLSECs

**Figure 9:**
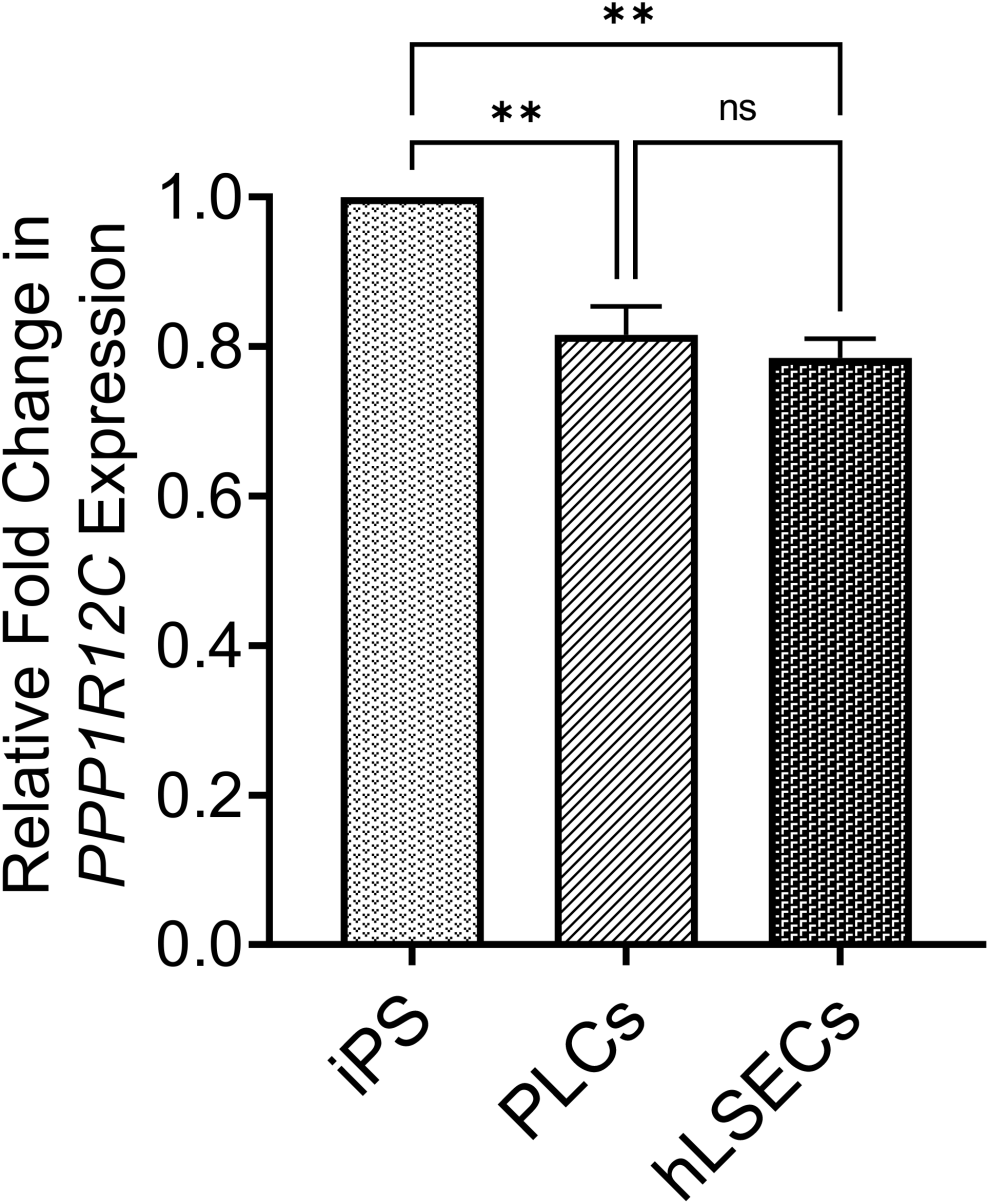
Graphical summary of expression levels of the *PPP1R12C* gene encoded by the AAVS1 locus to assess accessibility of locus for CRISPR/Cas9-mediated editing. RNA isolated from PLCs, hLSECs, and human iPSCs was subjected to RT-qPCR with primers specific to *PPP1R12*C and GAPDH as a reference. Relative fold-change in expression in PLCs and hLSECs is presented in comparison to human iPS cells after normalization with GAPDH as the housekeeping gene (n=3 for each cell type). Data are presented as mean plus/minus SEM; **p<0.01. ns, Not-significant.

As recent studies have shown that CRISPR/Cas9 can be used to introduce a GFP reporter into several different genetic loci (AAVS1 was not tested) in human mesenchymal stromal cells (MSC) (64), which are presumably similar to the mesenchymal PLCs used in the present studies, we performed studies to determine whether the accessibility/transcriptional activity of the AAVS1 genomic locus and/or the large size of the lcoET3 expression cassette might be responsible for the lack of CRISPR/Cas9-mediated insertion of the lcoET3 cassette into the AAVS1 locus of the PLCs and hLSECs. To address the first of these possibilities, we performed RT-qPCR on RNA isolated from human PLCs and hLSECs with primers specific to *PPP1R12C*, the gene encoded by the AAVS1 locus, comparing expression levels of this gene in these two cell types to that in human iPSCs, cells which are known to be highly amenable to CRISPR/Cas9-mediated gene knockin at the AAVS1 locus (65, 66). Relative levels of expression in each cell type were then calculated using the ΔCt method, with GAPDH serving as a reference. As can be seen in **Figure 9**, these studies revealed that the AAVS1 locus is significantly less accessible/transcriptionally active in both PLCs and hLSECs when compared to human iPSCs, providing a plausible explanation for why these cells might be more challenging to edit than iPSCs, but not explaining why they did not exhibit any editing.

**Figure 10:**
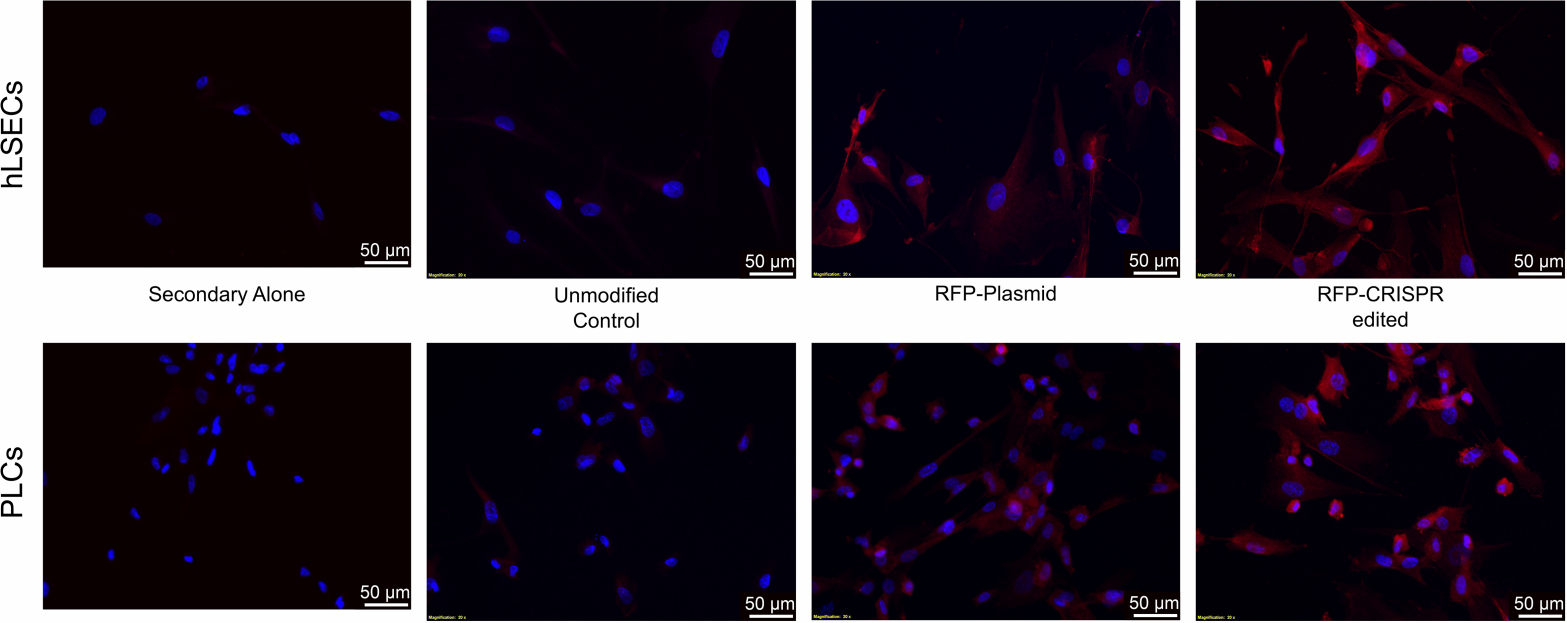
Immunofluorescence analysis of intracellular RFP protein in unmodified and gene-modified PLCs and hLSECs. PLCs (top panel) and hLSECs (bottom panel) in each experimental group were stained with a primary antibody for RFP that was then detected with an AlexaFluor^®^ 594-conjugated secondary antibody (red) and nuclei were counterstained with DAPI (blue). Controls consisted of slides with unmodified PLCs and hLSECs stained identically (labeled “controls”) and slides of each cell type stained with secondary antibody alone to establish levels of background fluorescence.

**Figure 11:**
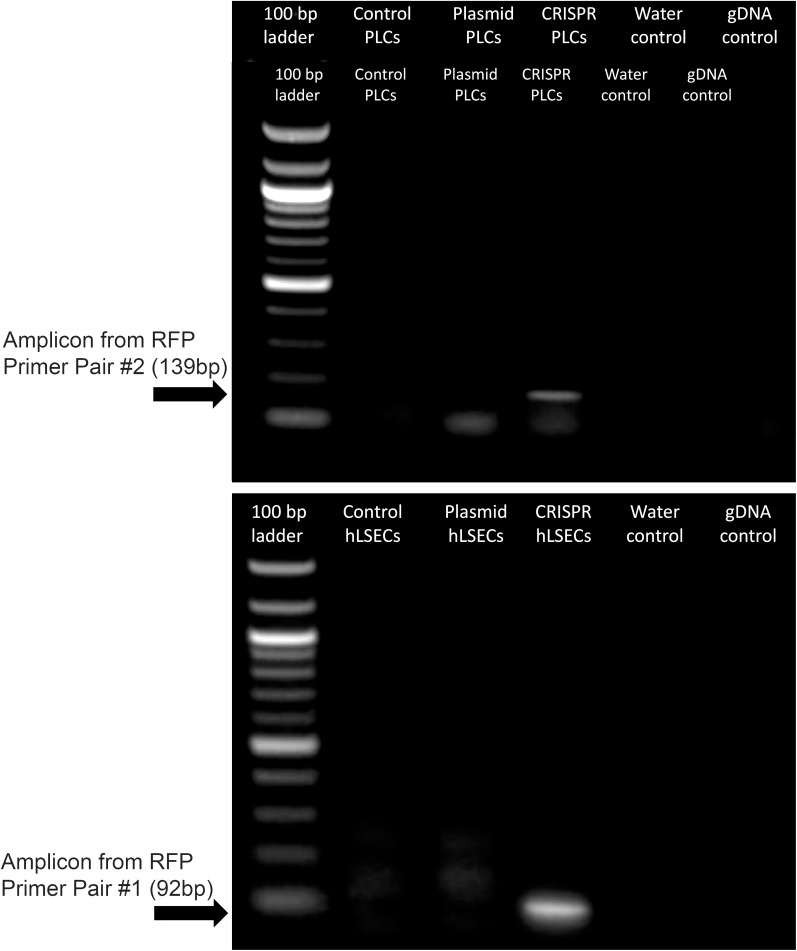
Expression of RFP in gene-modified PLCs and hLSECs by RT-qPCR. RNA was isolated from PLCs and hLSECs that were unmodified, transiently transfected with a plasmid encoding RFP, or had RFP permanently knocked-in *via* CRISPR/Cas9-mediated editing. RNA was isolated from the transfected cells after culture expansion and 3 passages and from the CRISPR/Cas9 edited cells following puromycin selection. RNA was then subjected to RT-qPCR and the resultant PCR products run on an agarose gel. Controls included no template/water and genomic DNA to confirm complete elimination of any contaminating gDNA by the RNase-free DNase step.

Another factor which could be precluding editing at this locus in the PLCs and hLSECs is the size of the lcoET3 expression cassette being inserted. Indeed, prior studies in human umbilical cord-derived mesenchymal stromal cells (MSC - PLCs are mesenchymal in nature) showed that a donor vector encoding GFP mediated far more efficient CRISPR/Cas9-mediated editing at the AAVS1 site than the identical donor vector encoding the marginally larger sRAGE gene (67). Given the very large size of the lcoET3 expression cassette (~5,600 bp), we performed experiments to address whether the cassette size was playing a role in the observed lack of editing. Specifically, we attempted to insert a red fluorescence protein (RFP) expression cassette (< ¼ the size of the lcoET3 cassette) into the AAVS1 locus of PLCs and hLSECs using CRISPR/Cas9. To this end, we performed experiments identical to those detailed above, co-transfecting PLCs and hLSECs with the pCas-Guide-AAVS1 plasmid (encoding both the sgRNA to the AAVS1 site and Cas9) and the pAAVS1-RFP-Puro-DNR donor template plasmid (the identical donor template plasmid used to achieve insertion of the lcoET3 expression cassette, but the lcoET3 expression cassette was replaced with a much smaller expression cassette encoding RFP). As in the experiments with the lcoET3 cassette, each cell type was also transiently transfected with the pAAVS1-RFP-Puro-DNR donor template plasmid alone. At 72 hours post-transfection (for the transient transfection groups; *n*=3 technical replicates for each cell type) and after selection in puromycin (for the CRISPR/Cas9-edited groups; *n*=3 technical replicates for each cell type), cells were passed to chamber slides and analyzed by immunofluorescence with an antibody specific to RFP. As can be seen in **Figure 10**, both transiently transfected and CRISPR/Cas9-edited, puromycin-selected PLCs and hLSECs exhibited robust staining for RFP, while unmodified PLCs and hLSECs did not, confirming successful uptake of the plasmid into both cell types and verifying that both cell types can be edited at the AAVS1 locus *via* CRISPR/Cas9. To further confirm CRISPR/Cas9-mediated editing of PLCs and hLSECs, RNA was isolated from an aliquot of each cell type at the time of plating for immunofluorescence and RT-qPCR performed with primers specific for RFP. The resultant PCR products were then run on an agarose gel and visualized with ethidium bromide. An image of the resultant gel appears in **Figure 11**, which clearly shows the correctly sized RFP amplicon in the puromycin-selected CRISPR/Cas9-edited PLCs and hLSECs, and its absence in both the unmodified and transiently transfected PLCs and hLSECs after 3 passages in culture. Collectively, these studies confirm hat the AAVS1 locus is a suitable target for gene knockin in both PLCs and hLSECs, and they support the conclusion that the large size of the lcoET3 expression cassette was likely the factor that precluded its knockin into this locus in PLCs and hLSECs.

## Discussion

As a monogenic disease, HA is an ideal candidate for correction by gene therapy. While current clinical trials are employing direct injection of AAV-based vectors (7–13), the use of gene-modified cells as vehicles to accomplish gene “addition” has many advantages from a manufacturing standpoint, as it allows multiple safeguards to be added to the production process (57). In an effort to make cell-based gene therapy a clinical reality for HA, we and others have performed studies over the past decades to identify the ideal cell type for delivering a fVIII transgene and the optimal vector to introduce the fVIII transgene into the desired cell population (3, 22, 31, 34, 37–48). Previously, we demonstrated the advantages of using lentivector-transduced PLCs as the cellular vehicle for delivering a fVIII transgene (22, 68). The present study evaluated whether CRISPR/Cas9 could be used to deliver an EF1α-lcoET3 expression cassette into PLCs, with the hope of further enhancing safety by directing integration to the AAVS1 “safe harbor” genome locus, thereby eliminating the theoretical risk of insertional mutagenesis that is inherent to integrating vectors such as those based upon lentiviruses. Furthermore, as the AAVS1 site is thought to be a transcripionally active site with open chromatic structure and native insulators that can resist transgene silencing, it stood to reason that the use of CRISPR/Cas9 to introduce the EF1α-lcoET3 expression cassette into this site might also enhance the levels of expression of the fVIII transgene and thus improve therapeutic efficacy (55). As there are thousands of different mutations spanning the whole FVIII gene locus that can cause HA (69), the use of this “knock-in” approach to insert a functional fVIII transgene into the genome, rather than trying to correct a specific HA-causing mutation, was deemed to be far more practical, as it would yield a universal treatment that could be administered to all HA patients. PLCs were simultaneosuly modified with two common modes of gene delivery, lentivector transduction and transfection with lcoET3-expressing plasmid, and side-by-side comparisons performed to results obtained with CRISPR/Cas9 gene-editing.

Although a good deal of progress has been made using CRISPR/Cas9-mediated gene editing to correct hemophilia B (HB), only a few studies have reported using this approach to attempt to correct HA (70–79), due in large part to the difficulty of achieving CRISPR/Cas9-mediated knock-in of a transgene with the increased length and complexity of FVIII (80, 81). The studies published to-date using CRISPR/Cas9 to correct HA have employed iPSCs, which enables selection of clones that have been edited successfully, which can then be differentiated into the desired cell types, such as endothelial cells or mesenchymal stromal cells (MSC) that can then be transplanted to mediate phenotypic correction (74–76, 82–87). The use of iPSC also has the added advantage of the ease with which these cells can be modified with genome-editing platforms, such as CRISPR/Cas9, that require induction of a doublestrand break (DSB) following by homologous recombination repair (HRR), a process that only occurs in actively dividing cells (88–92). The present study sought to determine whether it is possible to achieve similar success when the CRISPR/Cas9 system is used to insert a fVIII transgene cassette into a safe harbor in the genome of two primary, not iPSC-derived, cell types that are of direct clinical relevance to treating HA, human placental MSC (PLCs) (22) and human liver sinusoid-derived endothelial cells (hLSECs), the main cell type in the body responsible for synthesizing endogenous FVIII (93). In parallel, studies were performed using a lentivector and a plasmid to deliver this same cassette to PLCs and hLSECs to enable a side-by-side comparison of the efficacy of each approach.

The results reaffirm the potential of PLCs and hLSECs as cellular vehicles for delivering a fVIII transgene, showing both cell types can be transduced with a lentivector at high efficiency and subsequently express and secrete clinically meaningful levels of biologically active FVIII. Moreover, by using a bioengineered fVIII transgene (lcoET3) that contains elements from the porcine sequence that are known to facilitate ER trafficking (22, 33–36), these high levels of FVIII expression occur without induction of the stress response that can occur when misfolded FVIII accumulates in the ER (17, 33, 62, 63). This is an important finding, cells that are stressed as a result of over-expressing FVIII would not be predicted to engraft efficiently and survive long-term to provide sustained FVIII production and therapeutic effect. In addition, in similarity to what we previously reported in bone marrow-derived MSC (61), the use of lentivectors allows highly efficient gene delivery without evoking an innate immune response, as evidenced by the lack of expression of any of the TLRs in either PLCs or hLSECs following transduction with LV-lcoET3. Taken together, our results demonstrate that lentivector-mediated lcoET3 transgene delivery is a promising approach to engineer PLCs and hLSECs for cell-based fVIII delivery to treat HA.

In contrast to the results obtained with lentivector-mediated transduction, the transfection of plasmids designed to mediate the site-specific insertion of the EF1α-lcoET3 expression cassette into the AAVS1 genome locus *via* CRISPR/Cas9 revealed that primary human PLCs and LSECs are both highly refractory to such manipulation. Although endpoint PCR demonstrated the presence of DNA for the lcoET3 transgene in both cell types following attempts at gene-editing, neither cell type expressed appreciable levels of ET3 mRNA or protein, nor did they secrete significant levels of biologically active FVIII. Whole genome sequencing of PLCs followed by extensive bioinformatics analysis of the AAVS1 locus confirmed the absence of successful insertion of the EF1α-lcoET3 expression cassette at this site. The failure of CRISPR/Cas9 to mediate insertion of the EF1α-lcoET3 cassette is surprising given prior gene-editing successes by other groups with both FIX and FVIII (70–87) and prompted us to perform further studies to understand the factors responsible for the lack of successful knockin at the AAVS1 locus in the PLCs and hLSECs. The first factor we investigated for its possible role in the recalcitrance of PLCs to gene-editing was the site selected for gene insertion. While AAVS1 is considered to be a quintessential “safe harbor” locus, and has been used successfully in many studies, the accessibility of this locus in PLCs is unknown, and no gene expression data have been published that provide any hint as to whether the *PPP1R12C* gene encoded within the AAVS1 region is transcriptionally active in PLCs, or in MSC from any other tissue. Importantly, in a recent report (64) in which Cas9 was used to successfully insert PDGF-BB and VEGFA expression cassettes into MSC from bone marrow, adipose tissue, and umbilical cord blood, 3 different safe harbors were used for insertion, but not the AAVS1 site. To assess the basal transcriptional activity of this locus in PLCs and hLSECs when compared to iPSCs, which are highly amenable to gene knockin at this locus (65, 66), we performed RT-qPCR to quantitate expression of the *PPP1R12C*. These studies revealed significantly lower expression from this locus in PLCs and hLSECs, suggesting that the chromatin conformation in these cells may render them less amenable to CRISPR/Cas9-mediated knockin at this locus. Another contributing factor to the lack of gene editing in PLCs and hLSECs could be their markedly lower proliferative state when compared to the iPS cells that were employed in prior studies using CRISPR/Cas9 to knockin FIX or FVIII cassettes (70–87). Given that HRR only occurs in actively dividing cells (88–92), the PLCs and hLSECs used in the present study may not be ideally suited for HRR-mediated gene insertion, as their proliferation rate is markedly lower than that of iPS cells. This is especially true for the hLSECs, which exhibit very slow division kinetics and cannot be propagated for more than a couple of passages *in vitro* prior to senescing. The PLCs employed in the present study exhibit a phenotype and biological properties that closely resemble that of MSC from other tissues (3, 22, 68). It is noteworthy that prior studies have reported the downregulation of components of the DNA damage response (DDR) and HRR pathways in MSC with time in culture (94–96). As such, it is possible that the PLCs used in the present study, having been first cultured by explant, then selected for c-kit, and finally expanded for gene modification, no longer expressed sufficient levels of some of the key players in the HRR pathway required for efficient CRISPR/Cas9 gene-editing to occur.

Another key aspect that differs between the current report and prior studies in which MSCs were successfully edited with CRISPR/Cas9 (64, 67) is that the PDGF-BB, VEGFA, and GFP expression cassettes being inserted were substantially smaller than the EF1α-lcoET3 cassette employed in the present report. Prior studies have shown that it is far easier to achieve insertion of large inserts using NHEJ-mediated pathways than HRR (78). To ascertain whether the size of the EF1α-lcoET3 cassette played a role in the inability to achieve successful knockin at the AAVS1 locus in PLCs and hLSECs, we performed identical experiments using the same donor template, but replacing the EF1α-lcoET3 cassette with a much smaller CMV-RFP expression cassette. Both RT-qPCR and immunofluorescence data confirm that successful CRISPR/Cas9-mediated knockin of this smaller cassette can be achieved in both PLCs and hLSECs, supporting the conclusion that the large size of the EF1α-lcoET3 cassette was a major contributor to the apparent refractoriness of these cells to CRISPR/Cas9-mediated editing. One future avenue to explore would be to thoroughly characterize the PLCs and hLSECs with respect to their primary DNA repair pathways and/or to use cell cycle regulators such as Nocodazole and CCND1 (77) to induce specific pathways in an effort to improve the efficiency of CRISPR/Cas9-mediated knock-in of the very large EF1α-lcoET3 cassette into the AAVS1 locus of these cells.

It is also worth noting that the authors of the recent afore-mentioned MSC-editing study (64) used an AAV-based platform to deliver the gene-editing components, rather than transfection, as was employed in the present report. This likely improved efficiency of delivery of the gene-editing machinery. In addition, data presented herein demonstrate that the use of plasmids to deliver the CRISPR/Cas9 components led to dramatic up-regulation of TLR 3 and TLR 7 in PLCs, and it also triggered ER stress, as evidenced by upregulation of BiP. While the low levels of mRNA for the fVIII transgene argue that ER stress was not likely the cause of low secreted FVIII activity, the upregulation of ER stress is obviously not desirable, as it will likely negatively impact the viability and functionality of the PLCs, precluding their use in cell therapy. The upregulation of TLR 3 and TLR 7 by the plasmids would also likely compromise one of the key attributes of PLCs for use as an off-the-shelf therapy, namely, their state of relative immune-inertness, a conclusion supported by our recent report showing activation of PLCs leads to production of γ-IFN (68). As such, another lucrative avenue for future studies would be the redesign of the plasmids to remove any bacterial sequences that might be serving as pathogen-associated molecular patterns (PAMPs) and be responsible for recognition by the TLRs within the PLCs.

In summary, PLCs and hLSECs transduced with a lentivector encoding a bioengineered, expression/secretion-optimized fVIII transgene exhibit durable and robust FVIII expression and clotting activity without triggering innate immunity or ER stress molecules. Although the primary objective of inserting the lcoET3 transgene cassette into the AAVS1 site in PLCs and hLSECs *via* CRISPR/Cas9 was not achieved, the results presented herein provide mechanistic insight into the factors that precluded knockin of this cassette at this locus in these two cell types, and they validate the utility of both cell types as delivery vehicles for a fVIII transgene. Moreover, these data highlight the hurdles that remain to be overcome before primary human cells can be gene-edited with sufficient efficiency and the then be expanded to clinically relevant numbers for use in cell-based gene therapy to treat HA.

## Data availability statement

The original contributions presented in the study are included in the article/**Supplementary Material**. Further inquiries can be directed to the corresponding author.

## Ethics statement

The studies involving human participants were reviewed and approved by Institutional Review Board. The patients/participants provided their written informed consent to participate in this study.

## Author contributions

RR, MR, executed experiments, data analysis and interpretation; DM, AF, HCA, CL, CB provided technical expertise; CB, JS, and CB provided reagents; CBD, HTS, AA provided reagents and experimental feedback; RR drafted manuscript; GAP and CDP conception and experimental design, supervised experiments, wrote final version of the manuscript, and secured funding. All authors contributed to the article and approved the submitted version.
