# Supplementary figures and images for: Comparison of different gene addition strategies to modify placental derived-mesenchymal stromal cells to produce FVIII
Source: Front Immunol. 2022 Dec 15;13:954984. doi: 10.3389/fimmu.2022.954984 (PMC9800010; doi:10.3389/fimmu.2022.954984)

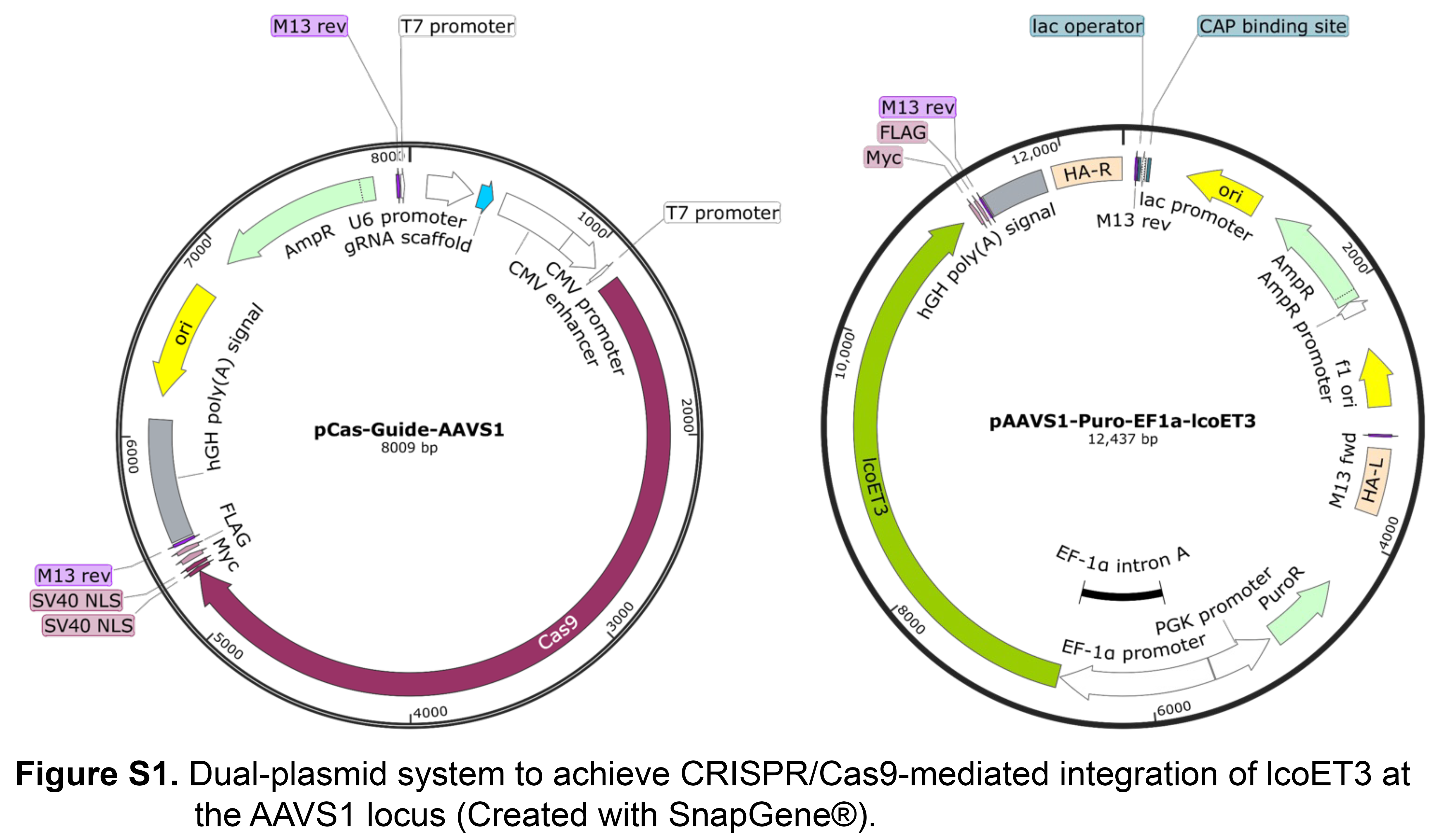

Supplement: Supplementary file 1 [file Image_1.tif]

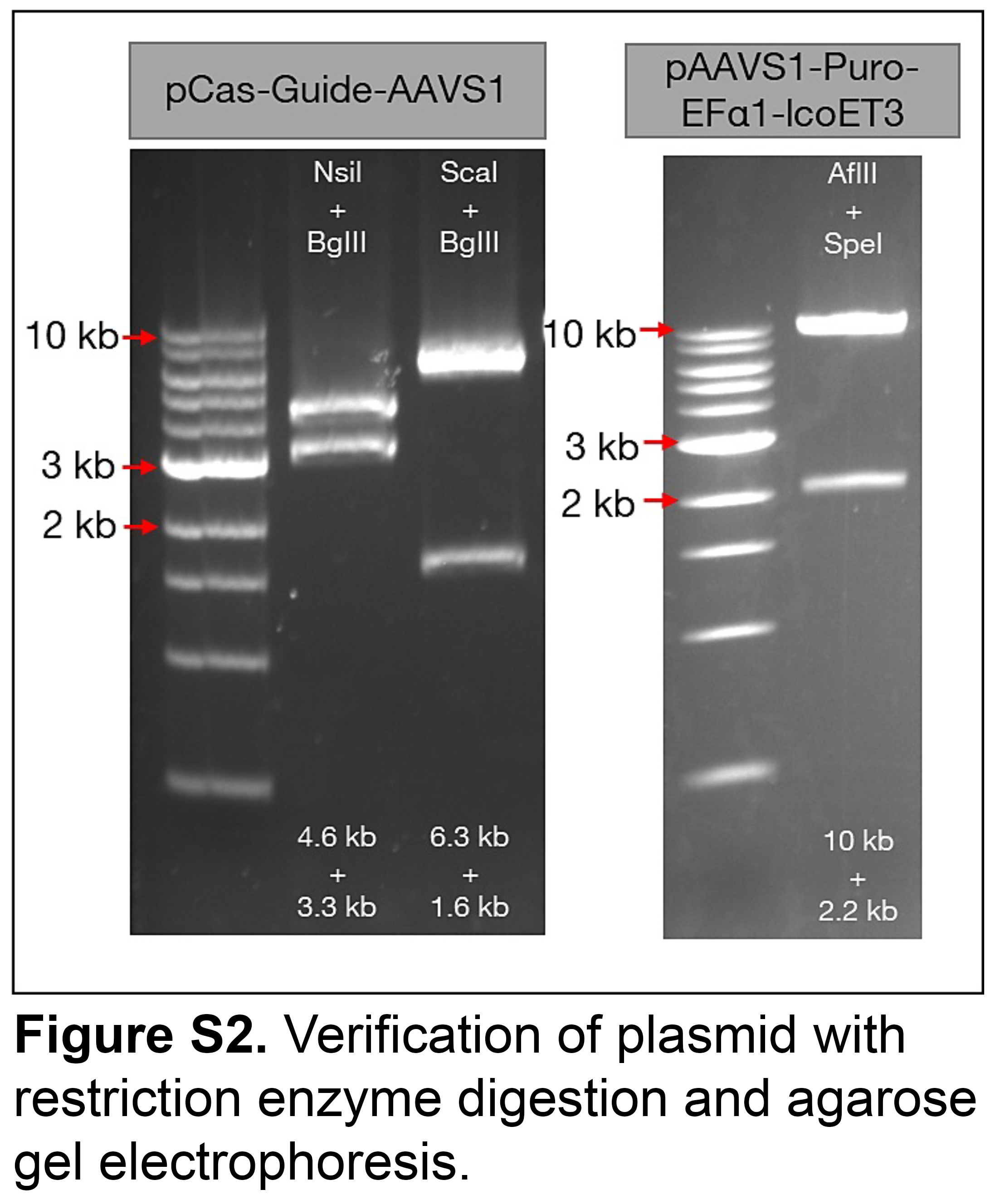

Supplement: Supplementary file 2 [file Image_2.tif]

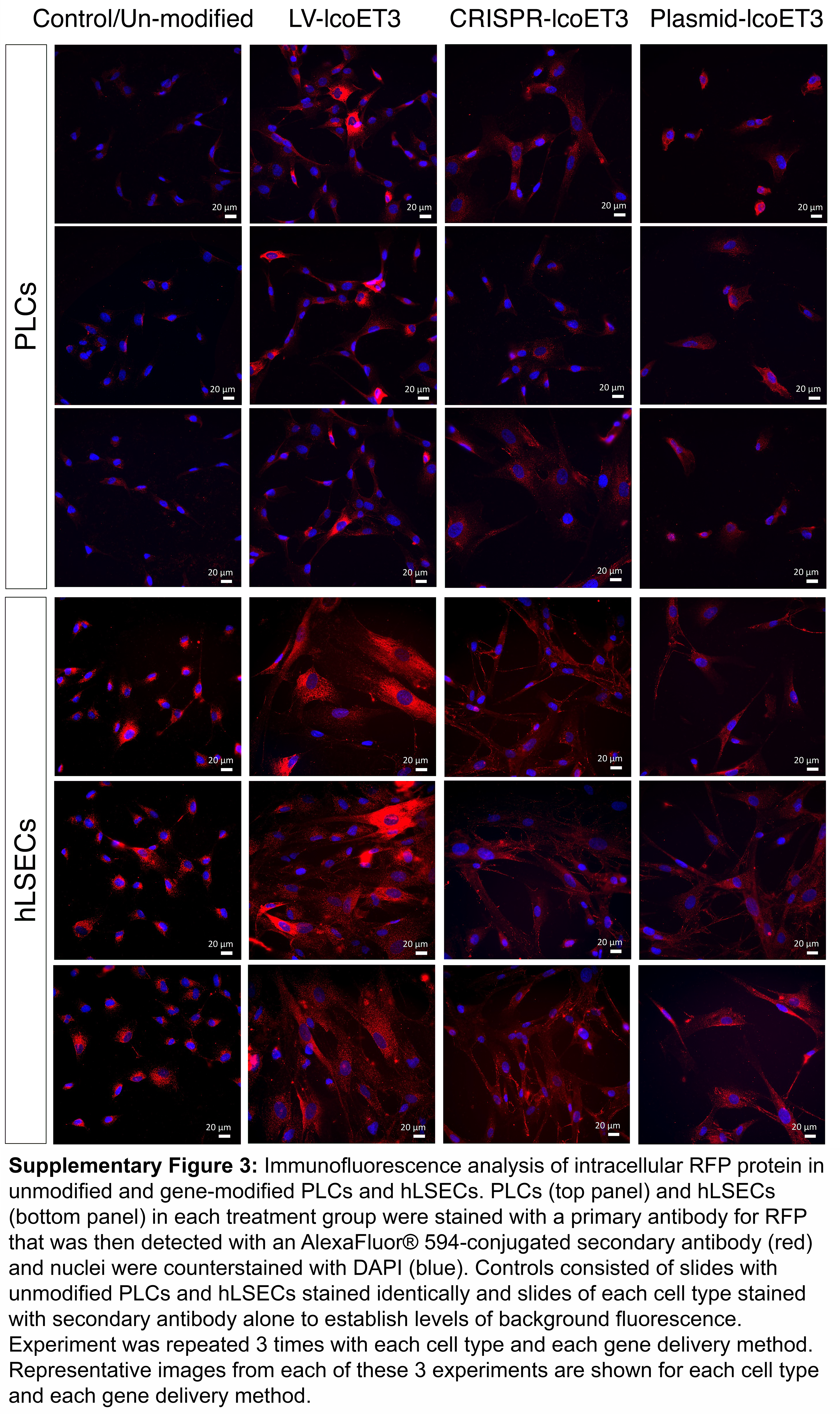

Supplement: Supplementary file 3 [file Image_3.tif]
